# Design and Validation of an Instrument to Evaluate Cognitive-Physiological Repercussions and Coping Strategies in a Pandemic Situation

**DOI:** 10.1007/s10728-025-00528-z

**Published:** 2025-06-10

**Authors:** Noelia Gallego-Ausín, María-Camino Escolar-Llamazares, Sandra Corral-Puente, María-Yolanda González-Alonso, Diego Serrano-Gómez, Andrea-Liliana Ortiz-González, Isabel Serrano-Pintado, María-Ángeles Martínez-Martín

**Affiliations:** 1 Department of Health Sciences, University of Burgos, Burgos, Spain; 2 Faculty of Psychology, Sergio Arboleda University, Santa Marta, Colombia; 3 Faculty of Psychology, University of Salamanca, Salamanca, Spain; 4 Department of Educational Science, University of Burgos, Burgos, Spain; 5 Gerencia de Atención Primaria, SACYL, Burgos, España

**Keywords:** COVID-19, Anxiety, Stress, Coping, Health personnel, University students, Professors

## Abstract

The coronavirus disease (COVID-19) pandemic has significantly affected mental health worldwide, highlighting the need for reliable tools to assess emotional impact (cognitive-psysiological repercusiones) and coping strategies. To validate the psychometric properties of an evaluation instrument with an ad hoc design, called the *Evaluation Inventory of Cognitive-Physiological Repercussions and Coping Strategies in a Pandemic Situation* (EICPCP), which has two scales: “Cognitive and Physiological repercussions arising from the pandemic due to COVID-19” (RCFPC19-13) and “Coping strategies during the pandemic due to COVID-19” (EAPC19-15). A total of 1,629 participants (students, teachers, and healthcare professionals) from Spain and Colombia completed the instrument. Exploratory and confirmatory factor analyses were conducted to assess their structure and reliability. Both exploratory and confirmatory factor analyses supported the five proposed dimensions: cognitive and physiological impacts on the RCFPC19-13 scale, and adaptation, support, and organization on the EAPC19-15 scale. In addition, satisfactory internal consistency, reliability indices, and temporal stability were obtained for both scales. The EICPCP is a valid and reliable tool for evaluating the cognitive-physiological impact of the pandemic and coping strategies employed. This instrument provides valuable insights for clinical research and psychological interventions in pandemic-related mental-health studies..

## Introduction

In the spring of 2020, the coronavirus disease (COVID-19) pandemic led to unprecedented lockdowns worldwide, profoundly disrupting daily life, the economy, and key sectors such as work, education, and healthcare ([84]; [81],Rek, Bühner, et al., 2020).

The pandemic has been associated with emotional disturbances, such as anxiety, fear, depression, post-traumatic stress, and even suicidal ideation, as documented in numerous studies [13, 23, 84],Magano et al., 2021; [82, 88],Rek, Bühner, et al., 2020; [111]. For instance, recent research has pointed out that the pandemic intensified psychological burden by exacerbating pre-existing vulnerabilities and disabilities [48], further compromising mental health and adaptation capacities.

In addition, the psychosocial impact was particularly severe among COVID-19 patients, where factors such as suicidal behavior, behavioral disorders, psychomotor agitation, migrant status, involuntary admissions, and private resident discharge were identified as significant predictors of psychiatric admissions following the lockdown periods [73]. Recent studies have shown that, following the lifting of lockdown measures, there was a significant increase in psychiatric emergency admissions, particularly for suicidal behavior and psychomotor agitation. This suggests the possibility of delayed mental health decompensation as a consequence of confinement, and highlights the importance of prioritizing access to mental health services during such transitions [5].

In the university environment, the temporary cessation of face-to-face activities during confinement and subsequent methodological changes have had a significant impact on students and professors’ lives and mental health [13, 20, 58]. Multiple studies have suggested that the sanitary alarm situation experienced by students and professors has caused an important threat to their emotional stability. Likewise, this situation has produced symptoms of anxiety and depression, among other mental health problems [35].

The pandemic has notably affected students’ learning and mental health, leading to increased stress, anxiety, and frustration due to academic overload and loss of personal control [2, 123]. Studies consistently have reported elevated levels of anxiety, depression, severe stress, and even suicidal ideation among students during confinement [42, 72, 112, 122, 127, 129]. For example, Odriozola-González et al. (2020) highlighted that both students and professors experienced mental health deterioration during confinement, with students showing the highest anxiety and depression levels.. Similarly, several studies have shown that the pandemic caused by COVID-19 and the measures implemented have had a negative impact on the mental health of Jordanian [27], American [108], Chinese (K. H. [124],Y. [125]), Arab [117] and Spanish [3] university students..

Given the high prevalence of emotional disorders among university students, the negative psychological impact of a pandemic on this population is expected [15, 54, 104]. Symptomatology is still reflected in college campuses [126].

Classroom closure forced professors to adapt their teaching to virtual formats without modifying the academic curriculum, sometimes under the threat of job loss [122]. These abrupt changes generate stress, anxiety, mood alterations, reduced resilience, and symptoms of post-traumatic stress (Q. [69, 71],Z. [33, 69, 71]). Healthcare personnel faced increased workload, long shifts [53], inadequate conditions, infection risks, and ethical dilemmas in resource allocation [97]. These factors, along with frustration, social stigma, isolation, fear of infecting relatives, and insufficient social support, severely impacted their emotional well-being [61, 78, 95]. This impact also extends to older adults and individuals with chronic illnesses, who have faced not only difficulties in accessing their usual healthcare services, but also an increased risk of emotional deterioration due to prolonged social isolation [6]. In such cases, remote mental health services have been suggested as a way to mitigate psychological consequences while also reducing the need for hospitalization [6]. These conditions exposed frontline healthcare workers to intense stress and psychological strain [61, 72, 120], often leading to physical and emotional exhaustion [22, 40]. The accumulation of these factors increased the risk of psychological distress and mental disorders [29, 92], including vicarious trauma linked to empathy towards patients (Q. [69, 71],Z. [69, 71]). Consequently, post-traumatic stress may persist as a long-term consequence [34, 46, 87, 97, 119].

In this context, coping strategies play a key role in mitigating the adverse effects of this collective trauma [31, 41, 104]. For instance, engaging in personal care and hygiene practices helps to reduce stress and prevent health problems [81]. Moreover, Eisenbeck et al. [31] showed that individuals with better initial mental and physical health coped more effectively with crises through proactive behavior, instrumental support, acceptance, humor, and personal meaning. Adopting hopeful attitudes, valuing life, and acting responsibly towards oneself and others were especially protective [31, 81, 85].

It is known that reliance on measures that are specific, reliable, and validated in this context can help professionals and public health authorities identify people at risk of experiencing adverse emotional reactions during and after a pandemic (de Medeiros et al., 2021; [113]). Likewise, knowing the coping strategies used is a key aspect in the search for factors that protect health [81, 85].

In the scientific literature, different scales are used to evaluate both the emotional consequences at the cognitive and physiological level, and coping strategies towards stressful events.

For example, to measure *emotional impact*, we found the *Vital Events Questionnaire Cuestionario de Sucesos Vitales* (Bonifacio [105]) through which vital events are evaluated in relation to various psychosocial areas, among which work, academic life, health, relations between partners, family, and children, social relations, residence, and economic and legal aspects.

Given the pandemic, new tools were developed to assess COVID-19-related anxiety. 63, 65, 66) designed the Coronavirus Anxiety Scale (CAS) and the Obsession with COVID-19 Scale (OCS). The CAS, with five items, measures dysfunctional anxiety (cognitive, behavioral, emotional, and physiological symptoms) and showed a unidimensional structure when validated in a U.S. sample [67]. High scores were linked to COVID-19 diagnosis, substance use, despair, and suicidal ideation [21],de Medeiros et al., 2021). Rehman et al. [101] created the NCAS (new coronavirus disease (COVID-19) anxiety scale), which assesses anxiety related to COVID-19 through three dimensions: threat, public policies, and societal consequences. The scale demonstrated good reliability (0.91) and incremental validity compared to instruments such as the FCV-19S and CAS, proving useful for identifying COVID-19-related anxiety. The OCS [65] detects clinically significant anxiety related to obsessive COVID-19 thoughts using four items. It identifies recurrent intrusive thoughts over two weeks and has shown good reliability and validity [21, 65]. Another instrument that contributes to the growing list of psychological evaluations at the time of COVID is the Coronavirus Stress Measure (CSM) developed by Arslan et al. [8]. It is a five-item instrument adapted from the Scale of Perceived Stress, the purpose of which is to measure the specific stress resulting from the COVID-19 pandemic, and it has adequate psychometric properties, constructs, and convergent validity [8, 57].

Rek, Bühner et al. (2020) likewise developed the COVID-19 Pandemic Mental Health Questionnaire (CoPaQ), which captures a wide range of pandemic-related psychosocial variables, including anxiety, stressors, mental health impact, conspiracy beliefs, and social cohesion. Despite its widespread international use, psychometric validation remains pending (Rek, Freeman et al., 2020).

The literature also includes various scales to assess *coping strategies*, defined as behavioral and cognitive efforts to manage internal or external demands perceived as overwhelming, depending on available resources [62]. These strategies aim to resolve stressful situations, providing relief, reward, or balance after a cognitive evaluation (Bonifacio [102, 103, 109]).

Among the scales used to evaluate coping, several were found. The Ways of Coping Scale by Sánchez-Cánovas et al. [101] stands out. This scale includes the eight dimensions of the Ways of Coping Inventory (WOC) of Folkman and Lazarus [39]: Confrontation, Distancing, Self-control, Acceptance of responsibility, Seeking social support, Escape-avoidance, Problem-solving planning and Positive reappraisal.

102, 103) Coping with Stress Questionnaire (CAE), which contains seven coping styles: Solution Focus, Negative Self-Focus, Positive Re-evaluation, Open Emotional Expression, Avoidance, Seeking Social Support and Religion.

The Coping Strategies Inventory (CSI) [ in its Spanish adaptation [19] with eight scales (problem resolution, cognitive restructuring, social support, emotional expression, problem avoidance, yearning, social withdrawal, and self-criticism).

The Academic Stress Coping Scale (A-CEA) by Cabanach et al. [18] which measures three strategies: Positive reappraisal, Seeking support and Coping through planning. The COPE-48 version [76] which collects nine coping strategies for stressful situations: Active coping, Alcohol or drug use, Focusing on emotions and venting, Seeking social support, Humour, Religion, Denial, Refraining coping, Acceptance, and personal growth.

Even so, despite the availability of specific instruments, we have found none with which we may evaluate the *emotional repercussions* (cognitive and physiological) generated by the COVID-19 pandemic and, at the same time, the *coping strategies* that the general population employ. As a consequence, the objective of this study was to develop and validate a simple inventory with two scales: emotional repercussions (cognitive and physiological), and coping strategies. This inventory was designed ad hoc to evaluate the psychological impact caused by the current pandemic and the coping strategies that were employed within a sample formed of students and teachers from Spanish and Colombian universities and Spanish health-care workers.

## Methods

### Participants

Data were collected from students and faculty members of the Universities of Burgos and Salamanca (Spain) and Sergio Arboleda and Metropolitana (Colombia) as well as from healthcare professionals in the Burgos Health Area (Spain). A total of 1,607 participants were included: 864 students, 229 teachers, and 506 healthcare professionals. The sampling was non-probabilistic and based on convenience. Recruitment was conducted online through institutional channels during April 2020, coinciding with the lockdown period in both countries.. In Tables 1, 2, the sociodemographic characteristics are detailed, adapted to the two large populations under study: on the one hand, university students and teachers and, on the other hand, health-care workers.

**Table 1:** Sociodemographic characteristics of the sample of students and teachers

			No. (%)
Characteristics	Total (%)	Student	Teacher
Population	1093 (100)	864 (79)	229 (21)
*Sex*			
Female	717 (65.6)	616 (71.3)	101 (44.1)
Male	376 (34.4)	248 (28.7)	128 (55.9)
*Age*			
< 25 years 25 – 35 years	689 (63.0) 167 (15.3)	687 (79.5) 115 (13.3)	2 (0.9) 52 (22.7)
> 35 years	237 (21.7)	62 (7.2)	175 (76.4)
*Nationality*			
Colombia	423 (38.7)	280 (32.4)	143 (62.4)
Spain	670 (61.3)	584 (67.6)	86 (37.6)
*Rural/Urban environment*			
Rural environment	214 (19.6)	194 (22.5)	20 (8.7)
Urban environment	879 (80.4)	670 (77.5)	209 (91.3)
*Living arrangements*			
Living alone	46 (4.2)	23 (2.7)	23 (10)
Accompanied	1047 (95.8)	841 (97.3)	206(90)
*Type of home*			
Own/family	846 (77.4)	671 (77.7)	175 (76.4)
Rented	247 (22.6)	193 (22.3)	54 (23.6)
*Civil state*			
Single	644 (58.9)	583 (67.5)	61 (26,6)
Married	151 (13.8)	41 (4.7)	110 (48.0)
In couple	269 (24.6)	236 (27.3)	33 (14.4)
Separated or divorced	23 (2.1)	2 (0.2)	21 (9.2)
Widowed	3 (0.3)	1 (0.1)	2 (0.9)
Living with a partner	3 (0.3)	1 (0.1)	2 (0.9)
*Knowledge areas*			
Health sciences	225 (22.7)	209 (25.9)	16 (8.6)
Social and legal sciences	513 (51.7)	396 (49.1)	117 (62.9)
Engineering and architecture	114 (11.5)	85 (10.5)	29 (15.6)
Sciences	53 (5.3)	47 (5.8)	6 (3.2)
Arts and humanities	88 (8.9)	70 (8.7)	18 (9.7)

**Table 2:** Sociodemographic characteristics of the sample of health care workers

Characteristics	No. (%)
*Sex*	
Men	87 (17.2)
Women	419 (82.8)
*Age*	
≤ 43	257 (50.8)
≥ 44	249 (49.2)
*Civil Status*	
Single	104 (20.6)
Married	265 (52.4)
Living with partner	107 (21.1)
Separated/divorced	28 (5.5)
Widowed	2 (.4)
*Level of studies*	
Doctor / Master’s degree	80 (15.8)
University studies: 1st and 2nd cycle degree	201 (39.7)
University studies: diploma	164 (32.4)
Vocational Training (Advanced)	37 (7.3)
Vocational Training (Intermediate)	24 (4.7)
*Work situation*	
Active	486 (96)
On Leave	5 (1)
Disability	12 (2.4)
Unemployed	3
*Working conditions*	
Permanent	215 (42.5)
Internship	141 (27.9)
Temporary	136 (26.9)
Resident	14 (2.8)
*Professional category*	
Facultative specialist	154 (30.4)
Nurse	257 (50.8)
Advanced technician	30 (5.9)
TCAE	34 (6.7)
L.E odontology	2 (.4)
Physiotherapist	10 (2)
Others	3 (.6)
*Work center*	
University hospital	336 (66.4)
First Aid	170 (33.6)
Urban	110 (21.7)
Rural	54 (10.7)

The data collection process was administered through an online questionnaire, which was circulated via email through different university bodies depending on the participating institution, in the case of Spanish and Colombian universities. And through the corporate email system of the Health Care Services of Castile and Leon (Spain), in the case of health-care workers.

The mailing was circulated in April 2020, that is, during the LockDown (LD) period. In particular, after four and three weeks of LD, respectively, in Spain and in Colombia.

### Instruments

**Socio-demographic questionnaire.** An ad hoc questionnaire was used to collect data on personal, academic, and occupational variables..

**Evaluation Inventory of Cognitive-Physiological Repercussions and Coping Strategies in a Pandemic Situation (EICPCP).** This instrument was developed ad hoc and was subject to validation to evaluate the psychological impact (at the cognitive and physiological level) due to COVID-19 and the principal coping strategies that were used. This instrument has a General Intellectual Property Registration Number: 765–1,015,161 (November 18, 2022). Registration was granted to the University of Burgos on behalf of the authors by the General Directorate of Cultural Industries, Intellectual Property and Cooperation, of the Ministry of Culture and Sports of Spain.

The EICPCP has two scales that can be either jointly or individually applied (Annex 1):

*Scale of cognitive and physiological repercussions arising from the pandemic due to COVID-19 ( RCFPC19-13)* []. This scale was used to evaluate the cognitive and psychological consequences associated with LD and the changing roles at work due to the COVID-19 crisis. It consists of 13 items grouped into two dimensions “Cognitive Repercussions” and “Physiological Repercussions”). The two dimensions, Cognitive and Physiological Repercussions, respectively, consist of 7 items and of 6 items. The items were evaluated on a Likert-type scale (1–6), where 1 corresponds to a few repercussions and 7 to many repercussions. A direct score between 13 and 34 points represents *repercussions,* between 35 and 56 points represents a *medium level of repercussion*, and between 57 and 78 points represents a *high level of repercussions*. The cut-off point for a high score was 57 (inclusive).The psychometric data were subjected to a validation process and are therefore specified in the results section.

*Scale of coping strategies during the COVID-19 pandemic (EAPC19-15)*. This scale evaluates coping in an attempt to understand how people manage the stressors that they face as a consequence of the changes in work and academic life arising from the COVID-19 pandemic. This scale consists of 15 items grouped into three related dimensions according to the way in which people manage the stressors: “Adaptation”, “Support”, and “Organization” (at a work and/or academic level). The three dimensions Adaptation, Support, and Organization have 5 items, 6 items, and 4 items, respectively. The items were scored on a 1–6-point Likert-type scale: 1 corresponding to total disagreement and 6 to total agreement. The range of direct scores was grouped in the following way: scores between 15 and 39 corresponded to *low levels of coping*; scores between 40 and 64 corresponded to *medium levels of coping*; and scores between 65 and 90 corresponded to *high levels of coping*. High scores were considered to be from 65 inclusively. Having been subjected to a validation process, the psychometric data are specified in the Results section.

### Procedure

Given the lack of instruments that accurately and adequately measure the variables: *emotional impact* (cognitive-physiological repercussions) and *coping strategies* in the COVID-19 situation, the process of developing the EICPCP instrument began. The EICPCP instrument was developed following the recommended stages for test construction [30, 89, 110].

The first phase included semantic delimitation of the constructs or domains and the development of items based on the bibliographic review and professional experience of the authors [110].

In relation to the construct-domain *emotional impact of the pandemic*, several scenarios were identified. According to Gómez Dávalos and Rodríguez Fernández [49], stress and concern in the era of COVID 19 can be defined from environmental, psychological, and biomedical perspectives. These three perspectives integrate stressful life events, subjective experiences, emotional response, and physiological systems involved. In this research, the domain *emotional impact* of COVID-19 was delimited based on the following aspects: a) Worry, tension, and stress in response to confinement; b) Concern, tension, and stress about the family; c) Worry, tension, and stress about social and emotional life; d) Worry, tension, and stress regarding the temporary uncertainty of obligatory physical isolation; e) Worry, tension, and stress in relation to contagion; f) Concern, tension, and stress about the economic impact; g) Worry, tension, and stress about the impact on their performance and/or decreased academic skills, such as concentration, memory, etc.; h) Worry, tension, and stress about work or academic workload.

Each of these eight aspects makes it possible to assess the emotional impact (cognitive-physiological repercussions) on university professors and students and health care workers during the confinement and the general COVID-19 pandemic situation.

In relation to the construct-domain *coping strategies during the pandemic*, it was established that adverse situations, such as confinement, could be difficult to accept. Crises such as those experienced by COVID-19, which affected a large part of humanity, allowed us to analyze whether the population, specifically university students and professors, and healthcare professionals, had coping resources.

Considering that coping strategies are used in each moment in a particular way and may vary depending on the triggering conditions, the aim is to measure how students, teachers, and healthcare workers cope with the challenges posed by confinement and the pandemic in general. For the assessment of coping, the following aspects were established, which would delimit the *coping strategies* constructed during COVID-19: a. Self-affirmation; b. Emotional expression; c. Social Support; d. Active coping. These four aspects guided the items that made it possible to assess the level of coping in university students and professors and healthcare workers.

Once the construct-domains have been defined on the basis of the theoretical foundation, the items that formed each scale were identified (P. [59]). The characteristics concerning the instrument (how it was to be administered, type of application, when it was to be applied, etc.) were determined previously.

The basic principles that were followed for the *construction of the items* were: representativeness, relevance, clarity, simplicity, and comprehensibility [89]. An initial *pool* of 41 items was grouped into two scales: Scale 1: Cognitive and Physiological repercussions arising from the pandemic due to COVID-19 (RCFPC19-13); and Scale 2: Coping strategies during the COVID-19 pandemic (EAPC19-15). These items were referred to a panel of experts composed of six (4 Spanish and 2 Colombian) professionals trained in clinical psychology and with broad experience in the field of mental health and 2 Spanish health professionals. As a result, some of the items were redistributed and others were removed.

With the resulting version, a pretest or pilot study was performed with a sample of 14 students, 8 teachers, 4 students, and 2 health-care professionals. The purpose was to examine the general functioning of the instrument in a sample of similar characteristics to the population that was under study [89]. The results served to improve the draft and to detect errors, not only in connection with the new instrument (EICPCP), but also in connection with a few questions related to some of the sociodemographic, work, and health-related questions (Socio-demographic Questionnaire).

Psychometric studies were performed on the resulting version (Annex 1), with the objective of contributing evidence pertaining to both the validity and reliability of the new instrument.

### Investigative Ethics

The project was developed considering all necessary measures to ensure compliance with all legal and ethical aspects of the investigation and the rights of the participants.

A favorable report was forthcoming from the Bioethics Committee of the University of Burgos (IR 11/2020) and Clinical Research Ethics Committee of Burgos and Soria (no. 2322). Likewise, compliance with international ethical criteria is contained in the Helsinki Declaration, Organic Law 3/2018, on December 5, Personal Data Protection and Guaranteeing Digital Rights (Spain), and Law 10/90 of September 6, 2006 (Colombia).

Both in the pretest (pilot study) and in the definitive sample, it was explained to the participants that they were to be identified with a code and that the confidentiality of their data was guaranteed. A database of any personal data was prepared for the study, in which the participants were identified with a code for each case. Having received the data, the information was reviewed and, if not coherent with the survey question, it was removed.

All participants provided their informed consent electronically before completing the questionnaire. The informed consent page presented two options (Yes/No). Only the participants who selected Yes were given access to the questionnaire, being free to abandon the process at any moment.

### Statistical Analysis

The degree of agreement among the experts on the quality of the items was assessed by means of Kendall's W coefficient. This coefficient provides content validity based on the concordance or agreement between the experts. A confidence level of 90% was established and a value greater than or equal to 0.50 was established as a criterion for retaining the item.

Subsequently, a preliminary analysis of the database was performed with the aim of analyzing the lost values, identifying the presence of atypical cases, evaluating normality, linearity, and the absence of multicollinearity between items, and testing the adequacy of the data for the factor analysis [43, 110].

The reliability of the instrument was evaluated through an internal consistency analysis of the items (Cronbach’s α) and bivariate correlations were calculated (Pearson’s r) for the analysis of the test–retest relation.

Subsequently, the internal structure of the instrument was evaluated through Exploratory Factor Analysis (EFA) and Confirmatory Factor Analysis (CFA) with two independent samples (n_1_ = 832; n_2_ = 770).

Finally, a structural equation model was prepared with a view to evaluating the statistical significance of the instruments under validation in relation to the absolute fit indices [Chi square, Root Mean Square Error of Approximation (RMSEA), Goodness of Fit (GFI) and Normed Fit Index (NFI)]; incremental fit indexes [Tucker Lewis Index (TLI), Comparative Fit Index (CFI), and Incremental Fit Index (IFI)]; and parsimonious fit indexes [Chi-squared ratio, Akaike Information Criterion (AIC), Conditional Akaike Information Criterion cAIC, and Adjusted Goodness of Fit (AGFI)]. CFI, IFI, TLI, GFI must remain over 0.90, AGFI must be over 0.80, and RMSEA must be under 0.08, for a well-fitted model. Cronbach’s Alpha coefficient was also estimated with the RFCPC19-13 and EAPC19-15 scales [91].

The criteria established in the literature were followed to determine the retention of the items in the factor analyses. In the exploratory factor analysis (EFA), items with factor loadings below 0.40 were eliminated, ensuring that only items with a significant association with their underlying factor were retained. In the confirmatory factor analysis (CFA), items with factor loadings equal to or greater than 0.70 were considered acceptable, indicating a strong relationship between the item and its latent construct. Factor extraction was performed using the principal components method with Varimax rotation, which allowed us to identify a two-dimensional structure that explained 59.23% of the total variance (31.62% for the first factor and 27.60% for the second factor). The first factor, called 'Cognitive repercussions', included items R1 to R7, while the second factor, 'Physiological repercussions', grouped items R8 to R13. Finally, the fit indices obtained in the CFA (CFI, IFI, and TLI above 0.90) confirmed the adequacy of the proposed two-dimensional model. These criteria and results guarantee the robustness of the factorial structure and the validity of the model.

IBM Statistics SPSS v.25 software was used to complete all analyses, except for the AFE used in the AMOS Graphics v24 (Analysis of Moment Structures) program.

## Results

### Selection of Items Agreement Experts

An initial pool of 41 items was prepared and sent to a panel of experts. Each expert was asked to evaluate the items on the criteria of clarity (whether the items are understood and whether their syntax and semantics are correct), coherence (whether they are logically related to the domain they measure), and relevance (each item is important and should be included). A response scale with four options (from 1 = strongly disagree to 5 = strongly agree) was used.

To determine the degree of agreement among the experts, Kendall's W statistical test was applied. Appropriate criteria were identified for each item of relevance for the two scales as shown in Table 3.

**Table 3:** Analysis of evaluation by criterion

	Clarity	Coherence	Relevance
Expert 1	2.86	2.86	3.40
Expert 2	3.63	3.63	3.61
Expert 3	4.44	4.44	4.24
Expert 4	2.61	2.61	2.58
Expert 5	3.54	3.54	3.46
Expert 6	3.93	3.93	3.71
W de Kendall	0.303	0.205	0.164
P	< .005	< .005	< .005

Based on expert evaluation, those showing a higher degree of inter-judge agreement were selected, comprising 28 EICPCP items covered in both scales: Scale of Cognitive and Physiological Repercussions arising from the pandemic due to COVID-19 (RCFPC19-13) with 13 items; and the Scale of Coping Strategies during the COVID-19 pandemic (EAPC19-15) with 15 items.

### Preliminary Examination of the Data

An initial exploration of the data was conducted, removing atypical cases and extreme values. Subsequently, the supposition of normality of the variables under study was verified [75, 94]. To do so, an analysis based on three strategies was completed: a) Graphic representation (Q-Q graphs), where the points situated on the diagonal line (Normal Q-Q Graph) and the randomness of the points situated around the horizontal line (Q-Q graph with no tendency), underlined that no normality problems could be found. b) Analytical methods where the asymmetry and kurtosis coefficients (Table 4) were found between -1/1, for which reason a normal distribution was considered [16].

**Table 4:** Description of sample normality

Scale	Statistic	Statistic
RFCPC19-13	Asymmetry	−0.083
	Kurtosis	−0.705
EAPC19-15	Asymmetry	−0.261
	Kurtosis	−0.110

And c) the Hypothesis Test (the Kolmogorov–Smirnov test and Lillefors correction of significance), yielding a significance level equal to 0.0001, with which the assumption of normality in this third strategy could be rejected.

Consequently, the results obtained using graphs and analytical methods confirmed the normality of the sample. In addition, the normality requirements may be reduced to the central limit theorem when the samples are sufficiently large [4], which was the case here, leading to the conclusion that the statistics required for the EFA have been fulfilled.

### Exploratory Factor Analysis (EFA)

Having completed the normality analysis, we tested whether the items were sufficiently interrelated [24] in accordance with each of the two scales. To do so, Bartlett’s sphericity test and the Kaiser-Mayer-Olkin (KMO) measure of sampling adequacy were performed. Before the analysis, 50% of the sample was randomly selected [7, 14, 28, 94].

The results of the *Scale of Cognitive and Physiological Repercussions arising from the pandemic due to COVID-19* (RCFPC19-13) led to the conclusion that it was reasonable to calculate the AFE, because the estimation of KMO = 0.925 (0.75 < *KMO* = 0.925 ≥ 0.5) yielded a satisfactory interrelation between the items [50]. It may be considered on the basis of Bartlett’s test of sphericity that there was sufficient correlation between the items, as *p* = 0.001 [32]. It was therefore feasible to conduct the AFE on this scale.

Having verified compliance with the basic assumptions, the factors were then extracted. The principal components method was used, which explained 59.23% of the total variance, composed of two factors. The first explained 31.62% of the variance and the second, 27.60% of the RCFPC19-13 scale [111]. It is recommended that the factor solution should be capable of explaining at least 50% of the variance explained by the factor structure [83].

The Varimax rotation method was used [56], yielding a rotated component matrix in which it was observed that Factor 1 (cognitive dimension) was composed of items from R1 to R7 and items from R8 to R13 were included in Factor 2 (physiological dimension). It was recommended that each factor be formed of at least 4 items with equal or higher correlations than 0.40, observing the highest item-factor correlations when grouping [47], which was the case of the proposed model.

In turn, adequate figures were obtained for the scale *Coping strategies during the COVID-19 pandemic* (EAPC19-15), with a KMO = 0.869 (0.75 < KMO = 0.869 ≥ 0.5) and the results of Bartlett’s sphericity test showed that the variables were sufficiently interrelated (*p* = 0.001).

The Principal Component Analysis (PCA) used as the extraction method yielded results that explained 57.02% of the total variance, obtaining a four-factor model. After the varimax rotation, a rotated matrix component was obtained in which Factor 4 was composed of only 2 items, for which reason the analysis was once again repeated, extracting three factors.

Upon completing the extraction of the 3-factor PCA, the model explained 50.19% of the variance. The first factor explained 22.15% of the variance, the second 14.80%, and the third 13.23%. Varimax rotation extracted a rotated matrix component in which Factor 1 (Adaptation dimension) was composed of items A1 to A5, Factor 2 (dimension supports) included items A6 to A11, and Factor 3 (dimension organization) included items from A12 to A15 (see the items in Annex 1).

It was concluded that the EFA proposed for the two scales—RCFPC19-13 and EAPC19-15 – was an adequate model for both, because the required assumptions, the extraction methods, the criteria with which to determine the number of factors and the factor rotation and interpretation methods were successful on both scales.

### Confirmatory Factor Analysis (CFA)

A database of 50% of the sample was randomly formed for the CFA [7, 14, 28].

**Fig. 1:**
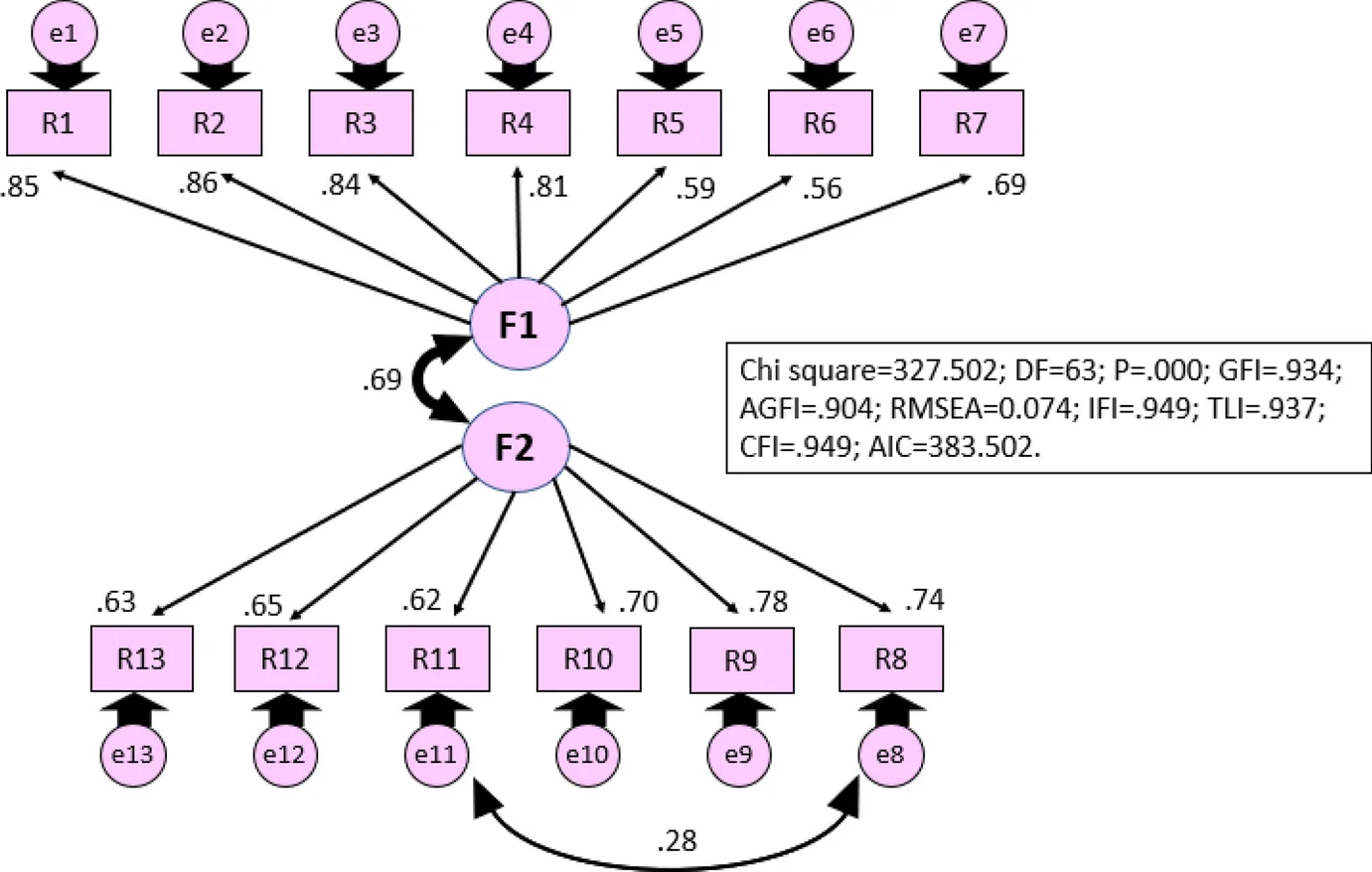
Bidimensional theoretical model on the RCFPC19-13 scale

As previously commented, the theoretical model used to produce the scale *“Cognitive and Physiological repercussions arising from the pandemic due to COVID-19” (RCFPC19-13)* was composed of two dimensions (cognitive and physiological repercussions). The Cognitive (F1) and Physiological (F2) dimensions consisted of 7 items and 6 items, respectively. The diagram shown in Fig. 1 was obtained when the theoretical model was represented in a graph.

Two latent factors may be observed in this model, which in our theoretical model are cognitive repercussions (F1) and physiological repercussions (F2), factors that were found to be interrelated. There were 13 variables to observe, the first 7 saturated the cognitive dimension and the last 6 saturated the physiological dimension. As may be appreciated in Fig. 1, each variable under observation is a saturated with a single factor. The measurement errors of the two variables under observation were found to be interrelated, with the purpose of improving the global fit. The estimated values were evaluated as a parameter that characterized the population through a sample. If it accurately represents the population, it is assumed that the statistic properly represents the parameter. The values estimated in the model must have a load ≥ 0.07 to be acceptable [10, 12], which was fulfilled in the model, as shown in Fig. 1.

The values that were obtained were evaluated from three perspectives: absolute fit, incremental fit, and parsimonious fit [52] (Table 5).

**Table 5:** Global fit indexes for the scales RCFPC19-13 and EAPC19-15

Models	*X*^*2*^	df	ECVI	RMSEA		NFI	GFI	AGFI	IFI	TLI	CFI	AIC	
RCFPC19-13	327.502	63	0.498	0.074		0.938	0.934	0.904	0.949	0.937	0.949	383.502	
EAPC19-15	335.480	85	0.527	0.062		0.899	0.944	0.922	0.923	0.904	0.922	405.480	

In relation with the absolute fit, *X*^*2*^_repercussion_ = 327.502 was obtained for 63 degrees of freedom with an associated freedom of less than *p* = 0.001. In this way, a ratio, *X*^*2*^/gl, was achieved that was not in accordance with the assumption of bivariate normality (*X*^*2*^/gl = 5.1 > 5).Therefore, Jöreskog [55] proposed the Root Mean Square Error of Approximation (RMSEA) by degrees of freedom. In this case, the result was 0.074 (RMSEA ≤ 0.1), indicating that the estimation of the parameter was not affected even though the assumption of bivariate normality was not fulfilled. In turn, RMSEA_repercussion_ = 0.74, IC90% = 0.066/0.082 showed a reasonable fit with the model, because it was ≤ 0.08, pointing to an approximation error of the model with reality (Bollen, 1989; Hu & Bentler, 1999; [60]).

The Expected Crossed Validation Index (ECVI) represents the correlation between the variables of the model, indicating reproducibility in different samples of the same population (ECVI_repercussion_ = 0.498, IC90% = 0.429/0.577), with results that were closer to 1, pointing to a higher correlation (R. [60]). The Akaike Information Criterion (AIC) and the Conditional Akaike Information Criterion (CAIC) are indexes that evaluate the parsimoniousness of the model (AIC_repercussion_ = 383.502 and CAIC_repercussion_ = 541.637). Their values were closer to those obtained for the saturated model than for the independent one and may be interpreted as indicators of a good global fit.

Among the measures of incremental and parsimonious fit, the Normalized Fit Index (NFI_repercussion_ = 0.938), the Incremental Fit Index (IFI_repercussion_ = 0.949), the Comparative goodness of Fit Index (CFI_repercussion_ = 0.949), the Tucker Lewis fit index (TLI_repercussion_ = 0.937), the Adjusted Goodness of Fit Index (AGFI_repercussion_ = 0.904), and the Goodness of Fit index (GFI_repercussion_ = 0.934) were all analyzed. These indexes may be interpreted as indicators of good incremental fit, because they exceeded 0.90 [11, 17], with which it may be concluded that the model represented the behavior of the data reasonably well.

**Fig. 2:**
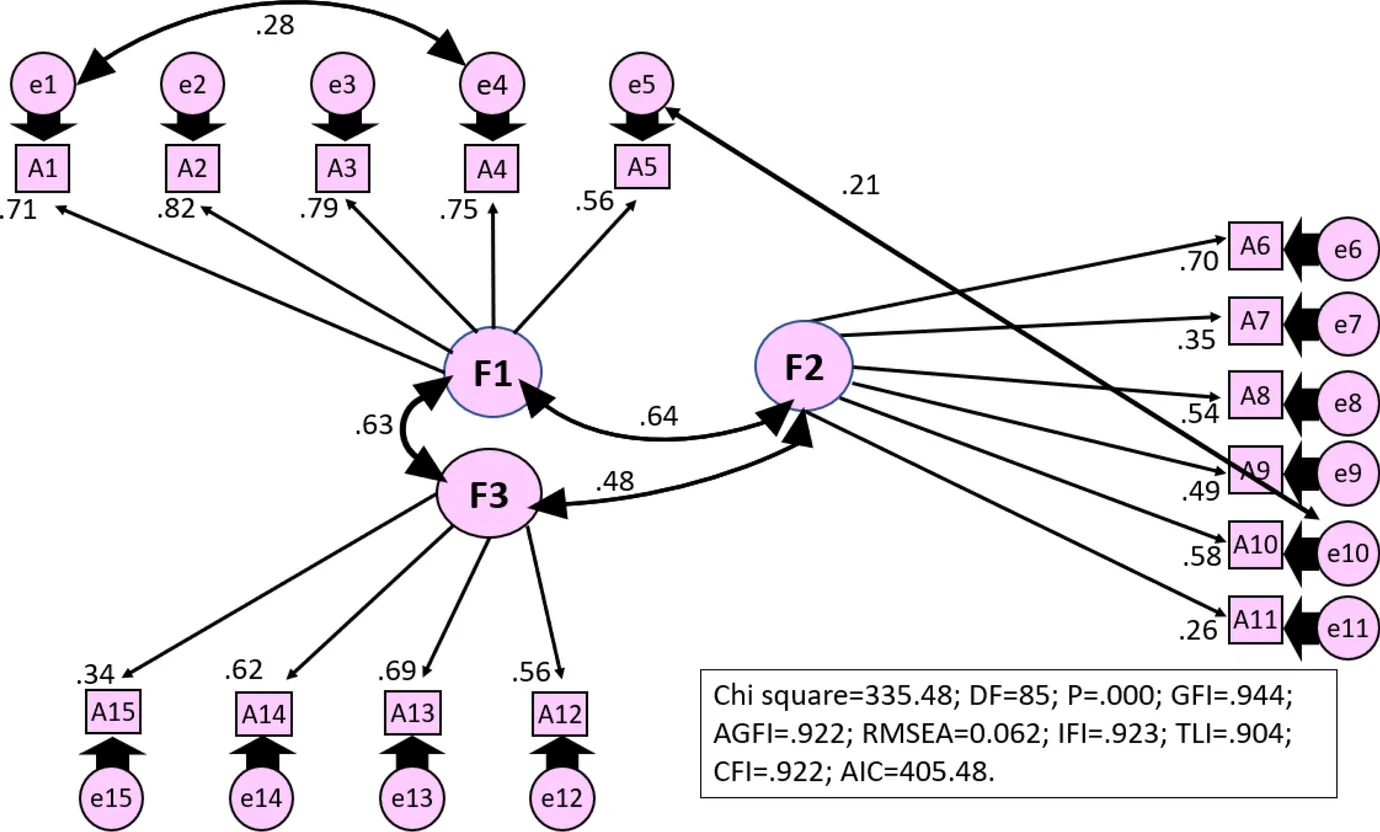
Theoretical tridimensional model on the EAPC19-15 scale

In the case of the scale *Coping strategy during the COVID-19 pandemic (EAPC1915),* the theoretical model had three dimensions (Adaptation, Support, and Organization) for its construction. The dimensions Adaptation (F1), Supports (F2), and Organization (F3) were composed of 5, 6, and 4 items, respectively. The diagram shown in Fig. 2 represents the theoretical model.

Three latent factors may be seen in this model, which in our theoretical model are Adaptation (F1), Support (F2), and Organization (F3), factors which were found to be interrelated. There were 15 observed variables, the first 5 saturated in adaptation, the following 6 saturated in supports, and the last 4 saturated in organization. As may be appreciated, each observed variable saturated a single factor. The measurement errors e1-e4 and e5-e10 of the observed variables A1, A4, and A5, A10 were interrelated between each other, with the aim of improving the global fit of the model. Therefore, the estimated values of a model may be considered acceptable, they must have a load ≥ 0.07 [10, 12], which the model fulfilled.

In relation to the absolute fit, the Chi-squared test yielded *X*^*2*^_coping_ = 335.480 for 85 degrees of freedom with an associated probability below 0.001. In this way, a ratio, *X*^*2*^/gl, was obtained, which fulfilled the assumption of bivariate normality (*X*^*2*^/gl = 3.9 < 5).

In Table 4, the list of fit indexes is shown referring to the EAPC19-15, which may be interpreted as a good fit of the model.

Finally, high general scores on the RCFPC19-13 scale indicated greater physical and cognitive repercussions arising from the COVID-19 pandemic. Gender and age had no effect on the pattern of responses on that scale. In the case of the EAPC19-15 scale, higher scores implied more coping strategies.

### Evidence of Reliability

In relation to the analysis of both reliability and internal consistency, highly satisfactory Cronbach’s alpha indexes for the two inventory scales of the EICPCP were obtained: α = 0.904 on the RCFC19-13 scale and α = 0.827 on the EAC19-15 scale (Table 6) [91, 93].

**Table 6:** Cronbach’s α of the coping (RCFPC19-13) and repercussion (EAPC19-15) scales due to the COVID-19 pandemic

	α
Repercussion (RCFPC19-13)	0.904
Physiological	0.846
Cognitive	0.896
Coping (EAPC19-15)	0.827
Adaptation	0.841
Supports	0.680
Organization	0.648

The temporal consistency and reproducibility of the (EICPCP) inventory with its two scales (RCFPC19-13 and EAPC19-15) that were created for health care workers were likewise evaluated*.* To do so, the instrument was administered at two different points in time, with an interval of one and two weeks [114], to a sample of 16 randomly selected participants, among health care professionals from the Burgos Health Area (Castilla y León, Spain).

Following the completion of the Interclass Correlation Coefficients (ICC), most items yielded statistically significant results (*p* ≤ 0.05) (except for item 9 of the RCFPC19-13 scale and items 3, 6, 7, 10, and 15 of the EAPC19-15 scale). According to the results, a high correlation could be established on the two scales CCI_RFCC19-13_ = 0.960 and CCI_EAPC19-15_ = 0.797, both with a high Interclass Correlation Coefficient > 0.75 [37, 38] (see Table 7).

**Table 7:** Test retest for the two scales: RCFPC19-13 and EAPC19-15

	CCI	*IC 95%*	*p*
R1	0.925	0.791/974	0.0001
R2	0.887	0.675/961	0.0001
R3	0.874	0.650/.955	0.0001
R4	0.826	0.499/.939	0.001
R5	0.883	0.673/.959	0.0001
R6	0.922	0.779/.973	0.0001
R7	0.923	0.781/.973	0.0001
R8	0.768	0.342/.921	0.004
R9	0.561	−0.325/.849	0.069
R10	0.901	0.723/.965	0.0001
R11	0.793	0.406/.928	0.002
R12	0.790	0.385/.927	0.003
R13	0.904	0.734/.966	0.0001
RCFC19-13 total	0.960	0.887/.986	0.0001
A1	0.800	0.413/.931	0.002
A2	0.747	0.300/.910	0.005
A3	0.472	−0.612/.820	0.124
A4	0.896	0.709/.963	0.0001
A5	0.828	0.506/.940	0.001
A6	0.570	−0.216/.849	0.058
A7	0.570	−0.269/.851	0.062
A8	0.939	0.825/.979	0.0001
A9	0.786	0.385/.925	0.003
A10	0.430	−0.737/.805	0.155
A11	0.802	0.439/.931	0.002
A12	0.573	−0.102/.845	0.041
A13	0.695	0.158/.892	0.013
A14	0.875	0.646/.956	0.0001
A15	0.416	−0.764/.799	0.164
EAPC19-15 total	0.797	0.416/.929	0.002

## Discussion and Conclusions

The situation caused by COVID-19 has meant a readaptation of the work methodology for both students and university professors. To the same extent, health personnel have been strongly affected in their work dynamics [45]. These changes at the academic and work level, together with the socio-sanitary circumstances of the pandemic, have had an important psychological impact on the university community and health workers [51]. The psychological impact must be known in order to establish prevention strategies. Therefore, it is essential to have validated instruments to measure students', teachers’, and health professionals' perceptions of the emotional impact of COVID-19 and the coping strategies developed. These results align with previous findings that highlight the disproportionate psychological burden experienced by vulnerable populations during the COVID-19 pandemic, particularly the elderly and individuals with chronic illnesses, who were more likely to suffer from isolation, reduced access to healthcare, and increased psychological distress [6]. This supports the need for targeted screening tools such as the EICPCP to identify and address the cognitive-physiological impact in these at-risk groups. In this regard, recent studies highlight that both individual and family functioning have been crucial in buffering the adverse psychological consequences of the pandemic, emphasizing the role of family cohesion, adaptability, and coping strategies [79].

Accordingly, the objective of the present study was to develop and to validate an instrument for the evaluation of emotional impact (cognitive and physiological levels) and coping strategies in a pandemic situation, as no instrument had been found in the literature that jointly measured both aspects.

To do so, items were prepared and selected based on expert evaluation and the relevant psychometric properties of the instrument were examined in a broad and heterogeneous sample (in age, geographical origin, occupation, level of study, and civil status…). An instrument (EICPCP) was obtained with two scales: RCFPC19-13 and EAPC19-15. The results confirmed that both content and construct validity of the instrument were adequate. In particular, the EFA showed a bifactor structure for the RCFPC19-13 scale (cognitive repercussion, physiological repercussion) and a tri-factor structure for the EAPC19-15 scale (Adaptation, Support, and Organization). This structure was validated through CFA on 50% of the samples that had been randomly selected [7, 14, 28]. These 5 factors represented five coherent and relevant dimensions from the point of view of emotional impact (cognitive and physiological level) and coping strategies towards the COVID-19 pandemic and made it possible to configure the two scales that comprise the EICPCP through 28 items with solid psychometric properties. High direct scores on the RCFPC19-13 scale indicate the higher cognitive and physiological impact of the COVID-19 pandemic. On the EAPC19-15 scale, higher direct scores implied more coping strategies. Gender and age did not affect the response pattern of these two scales.

Besides, the internal consistency of each dimension was satisfactory, with values that fluctuated between 0.648 and 0.896. Likewise, the corrected item-factor correlations suggested acceptable levels of temporal consistency (test–retest). A result that also guarantees the internal consistency of the two scales that constitute the EICPCP [25, 91]

The lack of valid instruments in the scientific literature that measure these five dimensions, grouped into two constructs: emotional impact and coping strategies in a pandemic situation, makes it difficult to compare information with other instruments. However, in relation to the *emotional impact construct* (cognitive and physiological repercussions), many instruments include these two dimensions in the measure of anxiety generated by COVID-19.

For example, the Fear of COVID-19 Scale (FCV-19S) by Martínez-Lorca et al. [121], based on the Fear of COVID-19 Scale (FCV-19S) by Ahorsu et al. [1], assesses fear of COVID-19 among Spanish students. The seven items that make up the FCV-19S relate to fear and anxiety reactions (heart rate, sweating, sleep disturbance, fear of dying, fear of infection, etc.). Items related to the physiological repercussions of our RCFPC19-13 scale. This indicates the importance of physiological changes in measuring anxiety responses. Similarly, Vally and Alowais [118], point out that the Coronavirus Anxiety Scale (CAS) [63, 68] focuses on the physiological symptoms of fear and clinical anxiety. For Vally and Alowas, this is one of the strengths of this scale.

Cognitive repercussions of the pandemic situation are also included in the main COVID-19 anxiety assessment instruments. In this sense, the CAS measures repetitive thoughts, preoccupation, and processing biases [68, 70], and the OCS (Obsession with COVID-19 Scale) [65, 66], 2020a) collects repetitive thoughts about COVID-19 and distressing thoughts. This finding supports the relevance of the cognitive repercussions of RCFPC19-13 scale.

Regarding *coping strategies,* there are no instruments that specifically measure this construct in a COVID-19 pandemic situation. Various scales are currently in use, most of which are based on the Ways of Coping Inventory (WOC) by Folkman and Lazarus [39]. The three dimensions of our EAPC19-15 scale (adaptation, support, and organization) can be identified in the main instruments measuring coping strategies: Coping Modes Scale by Sánchez-Cánovas et al. [101],Coping with Stress Questionnaire (CAE) by 102, 103); Coping Strategies Inventory by Tobin et al. [115], adapted by Cano García et al. [19] and Nava Quiroz et al. [90],Academic Stress Coping Scale (A-CEA) by Cabanach et al. [18],COPE-48 by Martínez Ortega et al. [77].

For example, the adaptation dimension of our EAPC19-15 scale appears in the above instruments through factors such as self-control, acceptance, and positive reappraisal. The support dimension is reflected in factors such as seeking social support, and the organization dimension in factors such as planning, focusing on problem solving, and active coping.

In conclusion, the results were satisfactory and demonstrated the reliable psychometric properties of the new instrument, providing a valid measurement tool to screen the emotional consequences of any pandemic situation and the principal coping strategies. Furthermore, studies focused on specific populations, such as pregnant women, have shown that contextual variables (e.g., partner support during delivery, restrictions, personal coping) significantly influenced emotional adjustment during the pandemic [26], reinforcing the need for tools such as the EICPCP, which assesses coping mechanisms. This aspect is of relevance because as Eisenbeck et al. [31] pointed out, the course of the current pandemic continues to be uncertain, with the appearance of new variants and the impact of protective measures it is still not over, as a consequence, we may be facing the start of a much longer, profound, and complex humanitarian crisis than whatever may a priori have been expected.

Likewise, in this study, we are responding to de Medeiros et al. (2021), who, after applying the CAS in their work, proposed a joint study as a future research of variables of both emotional nature and related to coping that were considered to protect against COVID-19-related anxiety. Along these same lines, Skalski et al. [107] pointed out the importance of considering other variables such as resilience and emotional support in future studies to combat COVID-19-related anxiety.

Taking these points into consideration, the EICPCP has been proposed as a useful tool, because it is a rapid, easy, and reliable way of knowing possible emotional moods and resources at an individual, group, and organizational level. Similarly, recent evidence indicates that university students' psychological well-being during the pandemic was directly affected by their coping resources and academic experiences, highlighting the need for instruments that capture both impact and coping dimensions [74]. In addition, the EICPCP offers the advantage, as with other scales such as the CAS, of separately evaluating emotional repercussions and coping strategies. This approach permits the development of more specific measures that increase the effectiveness of preventive strategies, interventions, and even public policies that are especially focused on people who feel vulnerable to troubled emotions that the COVID-19 pandemic is generating (de Medeiros et al., 2021; [86]).

The inclusion of samples from Spain and Colombia in this study allowed us to explore possible cultural differences in coping mechanisms and their impact on the validity of the EICPCP. Previous studies [9, 44, 96] have pointed out that sociocultural factors influence how individuals cope with stressful situations, with differences in the use of strategies such as seeking social support, positive reappraisal or emotional avoidance. In general, it has been observed that in Latin American contexts such as Colombia, coping tends to be more oriented towards social and religious support, while in European societies such as Spain, strategies focus more on problem solving and structured planning are favored [44]. However, despite these differences, psychometric analyses performed indicated that the factor structure of the EICPCP remains consistent in both samples, suggesting that the instrument is valid for assessing emotional impact and coping strategies in both cultural contexts. However, future research could delve into possible variations in the interpretation of the items according to cultural context to further improve the accuracy of the instrument.

The findings of this study have both theoretical and practical implications. First, the validation of the EICPCP confirms that it is a psychometrically sound tool for assessing the emotional impact of the pandemic in terms of cognitive and physiological repercussions, as well as the coping strategies used. These results suggest that the instrument may be useful for longitudinal studies analyzing the evolution of emotional response to health crises or prolonged stressful situations. Furthermore, our findings are in line with studies showing that the psychological effects of the pandemic did not end with the lifting of lockdown restrictions. In fact, Ambrosetti et al. [5] reported an increase in psychiatric emergency admissions due to suicidal behavior and psychomotor agitation in the post-lockdown period, underscoring the delayed mental health consequences of the pandemic. This further validates the relevance of tools like the EICPCP, which capture the ongoing emotional and physiological impact during and after crisis periods.

From an applied perspective, the EICPCP could be used in clinical and public health settings to identify risk profiles and develop targeted interventions to improve coping in the face of global crises. Furthermore, although the study focused on samples from Spain and Colombia, the factor analyses supported the structure of the instrument in different sociocultural contexts, which opens the possibility of adaptations in other Spanish-speaking countries. These findings contribute to the understanding of how people cope with the emotional impact of health crises and provide a valid tool to assess these processes in a structured and reliable manner.

Beyond the implications pointed out, some limitations of the present study must be highlighted. One of them is related with the sample that was not probabilistic (i.e., it was a convenience sampling.), which makes it impossible to generalize the results to larger populations that are shown here (de Medeiros et al., 2021). This means that although this approach allowed access to a diverse sample in terms of age and gender, it does not guarantee strict representativeness of the general population in Spain and Colombia. This limitation suggests the need to replicate the study in larger and more representative samples, using random or stratified sampling strategies to strengthen the external validity of the instrument. Despite this restriction, the psychometric analyses performed indicate that the factor structure of the EICPCP is stable, which supports its applicability in similar contexts.

It is likewise important to highlight the self-reporting implicit in the measure. Despite its presentation as a valid and reliable alternative to measure emotional problems and its associated factors, it is susceptible to biased responses [128] that are common to psychosocial research. Likewise, data collection was carried out at different temporal moments due to the administration needed to obtain permits and different restrictive measures specific to each country (Spain and Colombia). It must also be pointed out that transversal studies make it difficult to arrive at conclusions on the association of cause and effect [64]. This requires effort for the completion of longitudinal studies that will undoubtedly help to understand the future consequences in the short media, and long term of emotional implications and the principal coping strategies during a pandemic (de Medeiros et al., 2021; [80]).

Finally, it might be convenient to conduct new studies as future investigations that contribute additional evidence on the psychometric quality of the EICPCP, such as test criterion validity and discriminant and incremental validity with respect to other measures. Scales that especially evaluate anxiety, fear related to COVID-19, and coping strategies. In addition, alternative methodologies could also be considered in future investigations such as factor invariance (de Medeiros et al., 2021; [36]) with different groups (for example, country, gender, people with and without a diagnosis of anxiety). Doing so will help with the search for psychometric refinements that could reinforce the likelihood of detecting dysfunctional dynamics at an emotional level and coping strategies related with the pandemic (de Medeiros et al., 2021; [98]).

## Appendix

EVALUATION INVENTORY OF COGNITIVE-PHYSIOLOGICAL REPERCUSSIONS AND COPING STRATEGIES IN A PANDEMIC SITUATION (EICPCP)

### Cognitive and Physiological Repercussions arising from the pandemic due to COVID-19 (RCFPC19-13)

Indicate the level of repercussions that you have felt facing each of the following aspects of the current situation, on a scale of 1 to 6, where 1 is a LOW level and 6 is the HIGHEST level of repercussion.

**Table:**

COGNITIVE	1	2	3	4	5	6
**R1**: Level of concern, tension and stress that I am feeling with regard to lockdown						
**R2**: The lockdown situation is causing me and has caused me to feel concern, tension and family stress						
**R3**: I feel that this situation has caused concern, tension, and stress in my social and affective life						
**R4**: Thinking that this situation could repeat itself causes me to feel concerned, tense, and stressful						
**R5**: The crisis situation due to COVID-19 has caused me to feel concern, tension, and stress because of a fear of infection						
**R6**: The crisis situation due to COVID-19 is causing me to feel and has caused me to feel concern, tension and stress, because of a fear of the associated socioeconomic consequences						
**R7**: The situation of lockdown has caused me to feel concerned, tense and stressed, which influences my capacity for concentration, reducing my productivity						
**PHYSIOLOGICAL**	**1**	**2**	**3**	**4**	**5**	**6**
**R8**: I have felt emotionally overcharged with my work						
**R9**: During the health crisis, I have felt more tired than in normal situations						
**R10**: I have frequently suffered from headaches						
**R11**: The health situation which I have had to face has caused me to feel stressed						
**R12**: I have felt physically accelerated with hyperventilation and general agitation						
**R13**: I’ve been finding it more difficult to sleep deeply over recent months						

### Scale of Coping Strategies during the COVID-19 pandemic (EAPC19-15)

Read each one of the affirmations and mark the option that corresponds to your situation, on a scale of 1 to 6 where 1 means TOTAL DISAGREEMENT and 6 TOTAL AGREEMENT.

**Table:**

ADAPTATION	1	2	3	4	5	6
**A1:** I have been able to control the anxiety and the stress that the current crisis has generated within me						
**A2:** I have the capability to recognize my strengths and weaknesses without difficulty in this crisis situation						
**A3:** I consider that I have the resources to confront moments of crisis						
**A4:** In this crisis situation I can easily control my emotional reactions of happiness, sadness, anxiety and rage						
**A5:** When tension is generated in my work, I use conciliatory strategies that help to diminish stress						
**SUPPORTS**	**1**	**2**	**3**	**4**	**5**	**6**
**A6:** When I feel emotionally sad or demotivated these days, I have sought social support from my friends/companions/family members						
**A7:** I can easily place myself in other people’s shoes and this makes me suffer more than I might otherwise						
**A8:** I think that this situation that we are living through will make me stronger and will help me to value what is really worthwhile						
**A9:** Faced with negative thoughts and emotions of fear, sadness, I feel a desire to hug, give kisses…						
**A10:** I dedicate time to speaking with some friend/family members by telephone						
**A11**: The use of social networks helps to overcome difficult times						
**ORGANIZATION**	**1**	**2**	**3**	**4**	**5**	**6**
**A12:** I have been able to plan daily work in the best possible way						
**A13:** Even under these circumstances, I feel that I can easily create an agreeable climate with my companions						
**A14:** The feedback that I receive from people around me and from family members makes me feel better						
**A15:** I think that dedicating more hours to work/studies helps me to overcome the current situation						
